# Impacts of drought-tolerant maize varieties on productivity, risk, and resource use: Evidence from Uganda

**DOI:** 10.1016/j.landusepol.2019.104091

**Published:** 2019-11

**Authors:** Franklin Simtowe, Emily Amondo, Paswel Marenya, Dil Rahut, Kai Sonder, Olaf Erenstein

**Affiliations:** a International Maize and Wheat Improvement Center (CIMMYT), P.O. Box 1041-00621, Nairobi, Kenya; b Centre for Development Research (ZEF), University of Bonn, Bonn, Germany; c International Maize and Wheat Improvement Center (CIMMYT), Carretera México-Veracruz Km. 45 El Batán, Texcoco, C.P. 56237, Mexico

**Keywords:** Adoption, Weather risk, Drought tolerance, Yield, Crowding-In

## Abstract

Weather variability is an important source of production risk for rainfed agriculture in developing countries. This paper evaluates the impacts of the adoption of drought-tolerant maize varieties on average maize yield, yield stability, risk exposure and resource use in rainfed smallholder maize farming. The study uses cross-sectional farm household-level data, collected from a sample of 840 farm households in Uganda. The adoption of drought-tolerant maize varieties increased yield by 15% and reduced the probability of crop failure by 30%. We further show that the adoption of these varieties increased investments in maize production at the extensive margin through maize area increase and to a more limited extent at the intensive margin through mechanization. The findings show promise for further uptake and scaling of drought-tolerant maize varieties for increased productivity, reduced risk, and the transformation of the maize sector.

## Highlights

• A number of drought - tolerant maize varieties have been developed over the years.
• The adoption of drought -tolerant varieties is expected to increase productivity improve yield stability, and to reduce the exposure to risk.
• Drought-tolerant maize varieties also have the potential to empower producers to undertake risky, but high return investments.
• We apply an endogenous switching regression approach to assess how the adoption of drought tolerant maize varieties in Uganda affects such outcomes.
• We find that the adoption of drought-tolerant maize varieties increased yield by 15% and reduced the probability of crop failure by 30%.

## 1 Introduction

Agricultural production in developing countries is subject to various sources of risk (Anderson et al., 1977), with weather variability being a pervasive one (Hill et al., 2017). Such risks discourage farmer investments in productivity-enhancing technologies. Recent years have witnessed the development of stress tolerant crop varieties designed to help small-scale farmers manage weather stress. Drought Tolerant Maize Varieties (DTMVs) are one such promising avenue. These maize (*Zea mays*, also known as corn) varieties have an enhanced ability to withstand an abiotic stress like drought. A number of such DTMVs have been developed over the years in a collaborative effort by the International Maize and Wheat Improvement Center (CIMMYT) and National Agricultural Research Organizations (NAROs) particularly in Africa (Fisher et al., 2015). The DTMVs are screened in each of the countries where they undergo extensive on-station and multi-location on-farm testing using participatory variety selection approaches with farmers across the different agro-ecologies. The DTMVs that out-yield popular commercial checks in those agro-ecologies where they are tested are then selected for release and subsequent commercialization (Fisher et al., 2015; Setimela et al., 2017). By early 2016, over 200 distinct DTMVs had been released in 13 sub-Saharan countries, with reportedly more than 2 million farmers growing them (CIMMYT, 2017).

The adoption of stress-tolerant varieties is expected to increase productivity and yield stability, and importantly, to reduce the exposure of farmers to the downside risk. La Rovere et al. (2014) provided an ex-ante impact assessment of investments in drought-tolerant maize in Africa, based on the economic surplus method, and predicted positive yield impacts as well as improved yield stability. Ex-post empirical studies confirming these impacts in sub-Saharan Africa are still scant. One such study is Wossen et al. (2017), who point to the possibility of DTMV adoption having a yield stabilization as well as a risk reduction effect in Nigeria.

Risk-mitigating technologies such as DTMVs are thus expected to stabilize yields and incomes in the face of shocks, and possibly exhibit a risk reduction dividend. By reducing risks, DTMVs have the potential to catalyze investments in production and achieve higher incomes (Binswanger and Rosenzweig, 1993; Morduch, 1992). A risk reduction technology, therefore, can induce behavioral change, empowering producers to undertake originally risky, but high return investments, which they otherwise would avoid due to risk aversion. Accordingly, Dar et al. (2013) show how farmers with stress-tolerant rice seed increased their investments in rice fields. Via the risk reduction dividend, the adoption of DTMVs is expected to crowd-in additional agricultural investment at both the extensive margin (area planted) and the intensive margin (use of yield or value increasing inputs). Yet, the extent to which DTMV adoption crowds-in additional investments remains an empirical question. These are important empirical questions to better understand and refine the pathways of science-for-impact (Roling, 2009).

Against this background, the objectives of this paper are: (1) to assess how DTMV adoption affects mean productivity and variance, as well as exposure to downside risk; (2) to assess whether DTMVs adoption affects resource use. These are important issues to both confirm anticipated yield gains in farmers’ fields and understand farmers’ behavioral response. DTMV adoption may increase farmer investment at the extensive margin through the expansion of land planted with maize and/or the intensive margin through input intensification, and thereby have complementary impacts. Section 2 of the paper presents the conceptual framework. The data and the empirical strategy are presented in section 3. The results and discussions are presented in section 4, while section 5 concludes.

## 2 Conceptual and methodological framework

In this paper, we investigate the risk mitigating role of DTMVs, by adopting the moment-based approach proposed by Antle (1983) which enables the estimation of a stochastic production function under uncertainty. This approach to analyzing risk has also been applied by Wossen et al. (2017) to measure the impacts of adaptation strategies to drought stress in Nigeria and Di Falco and Chavas, (2009) to assess exposure to risk among farmers in Ethiopia. Theoretically, the approach assumes a production function comprising of outputs (e.g., yield), a vector of inputs including the DTMVs, weather-related variables which are exogenous and considered as the main source of risk as well as region specific fixed effects. Costs of production and prices are assumed to be non-random. Following Antle (1983), the component that captures the riskiness of a technology in a production function is estimated by exploring the behavior of the second (variance) and third moment (skewness) of the average yield of maize. While the mean output is expected to be strictly concave to inputs, the effects of inputs on the variance and the skewness of output remains an empirical question because an input could either increase, decrease or have no effect on variance (i.e., variance neutral) (Di Falco and Chavas, 2009).

This theoretical explanation is also premised on the fact that farmers maximizing the expected utility of net benefits from maize production will adopt improved technology if the expected utility from adoption is greater than the expected utility if they did not adopt. The expected utility of a risk-averse maize farmer can be further expressed as a function of all moments of the production function. Wossen et al. (2017) and Di Falco and Chavas (2009) provide a detailed account of the elasticity form of the production function, estimating the risk premium and the optimum conditions for adopting any technology.

### 2.1 The effects on productivity, variance, and skeweness

In the empirical estimation, we follow Lokshin and Sajai (2004) and Wossen et al. (2017) by applying an Endogenous Switching Regression (ESR) model to assess the causal effect of adopting DTMVs on yield, its variance, and skewness. A key assumption is that a specific farmer adopts DTMVs if the benefits expected from adoption are greater than those from non-adoption. Appendix 1.1 provides further details. The ESR applied also compares the expected outcomes (yield, variance, and skewness) of adopters of DTMVs (a) with respect to non-adopters (b), and to examine the expected outcome in the counterfactual hypothetical cases that the adopters did not adopt (c) and that the non-adopters adopted (d). Both the average treatment effect on the treated households (ATT) and average treatment effect on the untreated households (ATU) are estimated. Appendix 1.2 provides further details.

### 2.2 Effects on resource use

Following Emerick et al. (2016), DTMVs adoption can change the use of other inputs in three ways:

*Expected income effect:* DTMVs have a positive effect on expected income because they increase output in a drought year without compromising output in a year of good rainfall. This could increase input use in two ways (Emerick et al., 2016). First, progressive farmers could decide on input use levels based on expected returns. Second, increasing expected returns may increase risk-taking behavior. If farmers have preferences that exhibit decreasing absolute risk aversion, this would lead to intensified application of inputs.

*Marginal productivity effect:* Simple technical complementarity between the new DTMVs and an input would explain additional input use, because the adoption of the technology coincides with the increased return to the input used, an effect that arises independently of risk. In our case, there is a good reason to believe that DTMVs have an effect on the marginal product of inputs during both drought and good seasons as they were screened under both optimal and stressed conditions.

*Risk effect:* The adoption of DTMVs is expected to reduce the riskiness of input use. This downside risk effect is large when the new seed increases production in states of nature where the original marginal value of the input is low, and thus there are large losses from investing in inputs (Emerick et al., 2016). The implication in terms of the DTMVs is that spending on inputs may be more attractive to the farmer because the technology may still increase production even during a drought year, thus partially stabilizing consumption.

Appendix 1.3 provides further specification details.

## 3 Data and descriptive statistics

### 3.1 Data sources and survey design

**Fig. 1:**
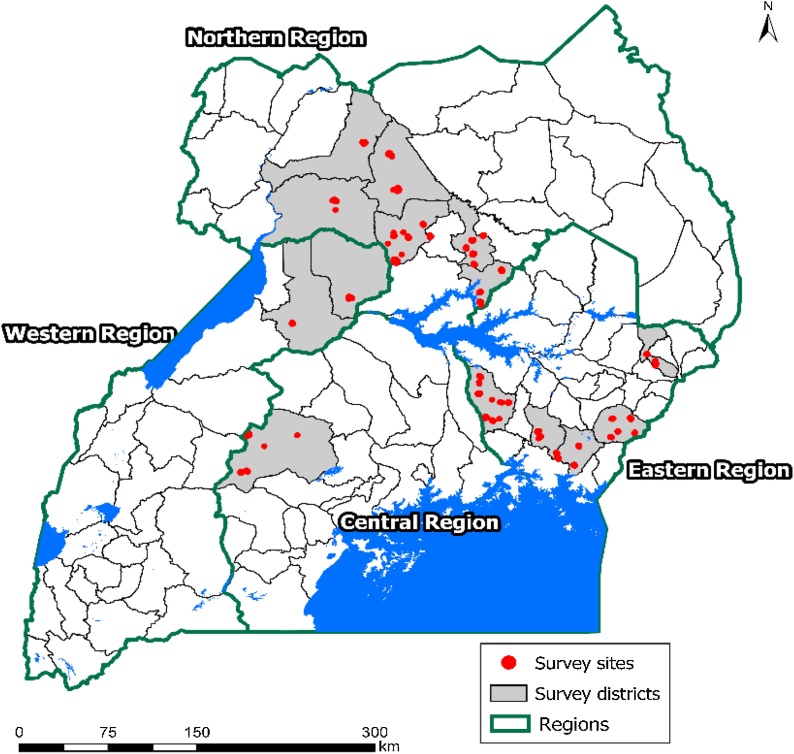
Survey locations in Uganda.

The data used for this paper draws from a survey of farm households conducted by CIMMYT in collaboration with Makerere University in Uganda in October 2015. The surveyed regions, districts, and households (Fig. 1) were selected using a multistage, random sampling technique.

The first stage involved the selection of four regions under the Feed the Future (FtF)^1^ zones of influence and where maize is widely grown. This led to the selection of 4 regions: Eastern, Western, Central and Northern. The second stage involved the selection of major maize growing districts from the four regions, which led to the selection of 14 districts. From each district, 42 villages were selected using a sampling design that makes explicit use of the population measure, “the probability proportional to size” sample design. About twenty households were randomly selected from each of the sampled villages. The total sample retained for the analysis amounted to 840 maize producing households, after dropping households (160) that did not grow maize in the major growing season of 2015.

The Eastern and Northern regions had larger samples because they are the largest maize producing regions in Uganda (Abate, 2013). From each of the selected households, detailed information was collected that included household demographic and socioeconomic characteristics, crop production, production conditions and utilization of maize, risk attitudes, food security, livestock ownership, and income categories of the household. Farmers listed the maize varieties that the household planted in the 2015 major growing season and how much land was allocated to each.

### 3.2 The diffusion and adoption process for DTMVs in Uganda

Uganda’s National Crops Research Resources Institute (NaCRRI) and National Agricultural Research Organization (NARO) in collaboration with CIMMYT developed DTMVs for Uganda. Since 2010, a number of DTMVs have been released in Uganda. After their release, seed delivery has been the responsibility of national agricultural research systems and public and private seed companies. Field demonstrations and field days have been used to diffuse^2^ information on the new DTMVs. NaCRRI and NARO played active roles in disseminating information about the varieties, channeling messages via posters, radio and television broadcasts, and newspapers (CIMMYT, 2014).

**Fig. 2:**
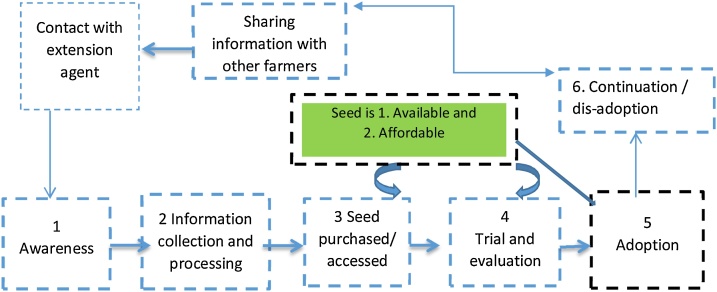
Stages in the farmer’s adoption process for improved seeds.

The adoption process of improved seeds is multi-faceted including multiple stages and various key influences (Fig. 2). In the case of DTMVs the adoption process starts with the potential adopter becoming aware of the existence of DTMVs. The second stage involves information acquisition, through which the potential adopter gets to know DTMV attributes and builds perceptions (Adesina and Forson, 1995). While this phase determines whether the producer has heard about the DTMVs, it is also a learning phase during which the potential adopter gets to further understand the attributes of a technology. Consistent with this notion, Klotz et al. (1995) posit that a producer's optimal information level is the solution to an underlying utility-maximization problem characterized by an income-leisure trade-off and that conditional upon the producer being aware of a new technology, the decision of whether to adopt the new technology is made. Technology information is a critical component in some of the recent adoption literature (Diagne and Demont, 2007; Simtowe et al., 2016; Kabunga et al., 2012) assuming that adoption is conditional on awareness. However, for farmers to move in to the third trial and experimentation stage also requires: (1) that seed is physically available and accessible (Dontsop et al., 2013); thus, seed is produced by a seed supplier and locally available; and (2) that seed is affordable to the farmer: thus, availed at prices commensurate with farmer’s incomes (Simtowe et al., in press). The seed must be available and accessible for adoption to take place. In Uganda, there are several seed companies involved in the production and marketing of DTMV seed. The fourth stage then involves trial or experimentation by the potential adopter on a small portion of land. The individual then goes through the fifth stage, which involves the actual DTMV adoption, which is again conditioned on the availability of and accessibility to the seed. After adoption, a farmer may decide to continue or discontinue using it depending on the experience and benefits.

### 3.3 Description of outcome variables

In measuring adoption, we captured the specific DTMV grown, the area cultivated with each DTMV and the year when they started growing the variety. This allowed us to capture both the names of the DTMVs grown as well as the intensity and history of cultivation for each of the DTMVs. We define a DTMV adopter here as a household that planted some DTMV on part or all of their maize fields in the survey year. In our sample, 14% of the households reported having planted at least one DTMV in one of their maize plots. Adoption was measured as a dummy variable taking the value of one for households that grew DTMVs, and zero otherwise. Some adopters did not fully allocate the land to DTMVs as they also continue to grow local and other improved maize varieties. The number of plots and the type of varieties planted on those plots by sampled households are reported in Table 1. The results show that about 13 percent of the maize plots were planted with DTMVs, with non-DTMVs comprising the remainder, including 48.6% other Open Pollinated Varieties [OPV], 12.4% other hybrids, while 26% were planted with local varieties. A total of 61% of the maize plots were thus planted with improved varieties that were non-drought tolerant.

**Table 1:** Types of maize varieties grown on sampled survey plots by region, Uganda.

Type of maize variety grown	# of plots by region				Total (n = 1069)	
	Eastern (n = 561)	Western (n = 55)	Northern (n = 383)	Central (n = 70)	#	%
DTMVs	101	16	20	3	140	13.1
Improved non-DTMV						
- OPVs	224	34	224	37	520	48.6
- Hybrids	76	1	32	22	131	12.4
Local	159	4	107	8	278	26.0

This study explores whether or not the adoption of DTMVs affects productivity, downside-risk and the crowding-in of other inputs. Hence the adoption decision is modelled as a binary variable both at plot and household level. Yields are based on farmer reported maize production per plot and plot size. Based on the yield, we computed two related dependent variables of interest: the variance and skewness of yield. The average yield for the surveyed households was 1.5 tons/ha (Table 2) which is slightly lower than the national average yield of around 2 tons/ha.

**Table 2:** Descriptive statistics by adoption status of drought-tolerant varieties, 2015 survey, Uganda.

	Households			Mean
	Full Sample (N = 840)	Adopters (N = 115)	Non adopters (N = 725)	difference
Household size	6.35	6.90	6.27	0.64^**^
Gender of household head (M = 1, F = 0)	0.87	0.87	0.87	0.00
Age of household head (years)	41.87	42.24	41.81	0.43
Years of education	6.47	6.68	6.44	0.24
Livestock herd (tropical livestock unit, TLU)	1.08	1.29	1.04	0.24^*^
Distance to market (km)	11.9	13.2	11.7	−1.44
Land holding size (ha)	1.78	2.22	1.71	0.51^**^
Maize area (ha)	0.49	0.64	0.47	0.17^***^
Received information on new maize (1 =yes)	0.39	0.53	0.37	0.16^***^
Group membership (1=yes,0 =otherwise)	0.79	0.82	0.78	−0.04
Hired labor (1 =Yes)	0.48	0.61	0.46	0.15^***^
Manure use (1 =Yes)	0.04	0.11	0.03	0.08^***^
Chemical fertilizer use (1 =Yes)	0.12	0.24	0.10	0.15^***^
Pesticide use (1 =Yes)	0.13	0.23	0.11	0.11^***^
Herbicide use (1 =Yes)	0.04	0.06	0.04	0.02
Fertilizer application rate (kg/ha)	7.84	14.01	6.86	7.15^**^
Grew at least one improved variety (1 =yes)	0.73	1.00	0.69	0.31^***^
Seed rate use (kg/ha)	26.04	21.82	26.71	−4.89^***^
Decision making by head (1 =yes)	0.52	0.48	0.52	−0.04
Decision making by spouse (1 =yes)	0.10	0.06	0.11	−0.05
Good soil fertility plots (1 =yes)	0.63	0.65	0.62	0.03
Slope is steep (1 =yes)	0.10	0.09	0.10	−0.01
Slope is moderate (1 =yes)	0.32	0.40	0.31	0.09^*^
Yield of maize (kg/ha)	1548	1731	1519	212^*^
No erosion (1 =yes)	0.69	0.60	0.71	−0.11^**^
Irrigation use (1 =yes)	0.02	0.02	0.02	0.00
Income indicators:				
- Income allows to build savings (1 =yes)	0.10	0.17	0.09	0.09^***^
- Income allows to save a little (1 =yes)	0.37	0.43	0.36	0.06
- Income = expenses (1 =yes)	0.29	0.19	0.30	−0.11^**^
- Insufficient income, use savings to meet expenses (1 =yes)	0.16	0.17	0.16	0.01
- Insufficient income, borrows to meet expenses (1 =yes)	0.08	0.03	0.09	−0.05^*^
Eastern region (1 =yes)	0.50	0.72	0.47	0.26^***^
Western region (1 =yes)	0.05	0.11	0.04	0.07^***^
Northern region (1 =yes)	0.38	0.15	0.41	−0.26^***^
Central region (1 =yes)	0.07	0.02	0.08	−0.06^**^

^*^,^**^,^***^ imply the difference in significance at 10%, 5%, and 1% levels, respectively.

**Fig. 3:**
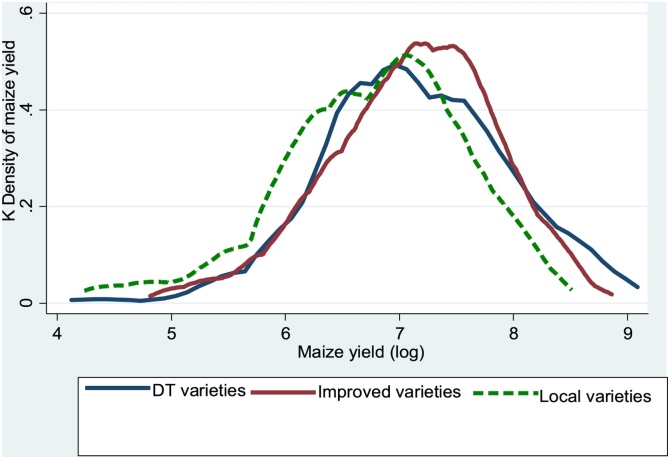
Distribution of Maize yield, for DT, Improved non-DT, and Local varieties, 2015 survey, Uganda.

Fig. 3 reports the distribution of plot-level maize yields for DTMVs, improved non-DTMV, and local varieties. The yields were higher for DTMV and other improved varieties compared to local varieties. The yield distribution for local maize growers is more skewed to the left than the yield distribution for DTMV growers, which suggest that growing DTMVs significantly reduces the probability of crop failure. Moreover, a two-sample Kolmogorov-Smirnov test for equality of distribution functions revealed significant differences (at 1% level) in the distribution of yield functions between local varieties and DTMV, whereas the differences in the distribution functions between DTMVs and other improved maize varieties were not significant.

The distribution functions also suggest that the probability of crop failure from growing local varieties is much higher than the probability of crop failure from growing DTMVs and other improved non-DTMVs. This shows that the benefits from growing DTMVs relative to local varieties are twofold: (i) they offer higher yields and (2) they offer gains from risk reduction through the reduction in exposure to downside risk.

### 3.4 House socio-economic and plot characteristics

Table 2 presents descriptive statistics for some of the explanatory variables used in the analysis disaggregated by the adoption status of the households. About 87% of the households were male-headed. Households were drawn from Eastern (50%), Western (5%), Northern (38%) and Central (7%) regions of Uganda, with Eastern and Western regions having a significantly higher proportion of adopters than non-adopters.

An average household consisted of 6.4 persons, with adopting households reporting significantly (at 5% level) larger households (6.9 persons) than the non-adopters (6.3 persons). The average land holding size was 1.8 ha and adopting households had significantly larger landholdings (2.2 ha) than the non-adopters (1.7 ha). Average land allocated to maize (0.49 ha) accounted for 28% of the total land, with adopting households allocating significant more area to maize (0.64 ha) compared to non-adopters (0.47 ha), an issue revisited below in relation to the regression results for resource effects. Similarly, the adopters had significant larger livestock herds (1.29) as compared to non-adopters (1.04).

Information on household income categories was also collected. On average, the majority of households were in the income category that allowed them to save a little (37%). It was also noted that adopters dominated the top income category - that allowed to build savings (17%) as compared to 9% of non-adopters. To capture access to information, farmers were asked whether or not they received information about new varieties. About 39% of the sampled households reported receiving information about new maize varieties in 2015, being more common for adopters (53%) than the non-adopters (37%). This suggests that the access to information on new maize varieties affected the likelihood of cultivating at least one DTMVs and non-adopters were more information constrained than adopters.

In terms of input use, adopters were more likely to use hired labor, manure, chemical fertilizers, and pesticides. Adopters also reported higher maize grain yields (1.7 ton/ha) as compared to non-adopters (1.5 ton/ha). Overall, DTMV adopters appear to be better endowed, cultivate more maize and have higher input use and yields.

Rainfed maize production in Uganda remains relatively extensive, with limited external input use. For instance, fertilizer use was reported by 12% of the surveyed households with an application rate of 8 kg/ha. This compares to findings by Namaazi (2012) who report annual inorganic fertilizer application rates in Uganda of about 2 kg/ha. Namaazi (2012) also reports that relatively modest investments by smallholders in inorganic fertilizer could dramatically increase their maize productivity in Uganda and that medium rates of fertilizer application (60 kg/ha) yielded a 270 percent increase in yield over no fertilizer application.

**Fig. 4:**
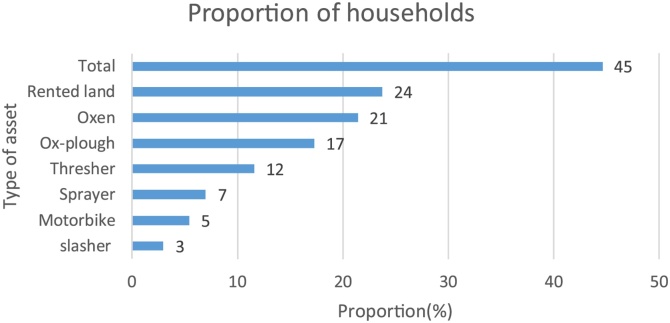
Household hiring of assets /implements for maize farming, 2015 survey, Uganda.

The hiring of assets was quite prevalent in the study regions, with about 45% of households reporting hiring some implements/assets for their farming activities (Fig. 4). The most frequently hired asset was land (24%), perhaps indicating its scarcity, as well as the potential market for land. Hiring oxen and ox-plough for land preparation were reported by 21% and 17% of the households, respectively. This suggests that most land preparation was done using the hand hoe. The finding is consistent with other studies (FAO and UNIDO, 2008) which show that farm power in African agriculture relies heavily on the hoe and other hand tools, with 65% of cultivated land in sub-Saharan Africa prepared by hand, 25% by draught animals and 10% by tractor (FAO, 2013). The hiring of post-harvest equipment, such as the thresher, was reported by 12% of the households while the use of a hired sprayer was reported by 7% of the households.

## 4 Results and discussions

### 4.1 Impacts of drought-tolerant maize adoption on productivity and risk exposure

#### 4.1.1 Impact on productivity

Table 3 presents the expected land productivity (i.e., maize yield per hectare) under actual and counterfactual conditions. Cells (a) and (b) represent the average observed sample yields (in log form) for adopters and non-adopters, respectively. At first glance, the observed yield by DTMVs adopters is higher than the non–adopters, but this simple comparison of observed mean yield without controlling for other factors that might influence productivity differentials between adopters and non-adopters can be misleading. The correct unbiased comparison is cells (a) vs (c) and (b) vs (d) in Table 3 (and Appendix 1) which controls for both observable and unobservable farmers characterics that influence yield. The results confirm that DTMV adoption has a positive impact on yield for adopters. It shows that the treatment effect for DTMV adopters is +0.96 which is equivalent to a 15.4% increase over the average yield. However, the yield of non-adopters would have been reduced by 2% if they had adopted DTMVs.

**Table 3:** Estimates of the effect of DTMV adoption on maize yield, 2015 survey, Uganda (Endogenous Switching Regression model, ESR)^a^.

Outcome variables	Household type and treatment effect	Decision stage		Effect on adoption	Change (%)
		To Adopt	Not to adopt		
Log of average maize yield:		(1)	(2)	(3)	(4)
1. DTMV vs all non-DTMV [improved or local]	Adopters (ATT)	(a)7.18	(c)6.22	0.96^***^	15.4
	Non-adopters (ATU)	(d)6.94	(b)7.08	−0.14^***^	−2.0
	Heterogeneity effect	BH1=0.24	BH2=-0.86	TH=1.1	17.4
2. DTMV vs improved non-	ATT	7.18	6.3	0.88^***^	14.0
DTMV	ATU	6.94	7.11	−0.17^**^	−2.4
	Heterogeneity effect	0.24	−0.81	1.05	16.4
3. DTMV vs local	ATT	7.18	6.04	1.14^***^	18.9
	ATU	6.94	6.98	−0.04	−0.5
	Heterogeneity effect	0.24	−1.07	1.31	21.3

^***^p < 0.01, ^**^ p < 0.05.

a We include controls in the switching regression such as education, gender, age, household size, farm size, drought shock, use of fertilizer, manure, herbicides and other chemicals, membership in a group as well as plot characteristics such as soil quality as well as the slope. Location dummies include Eastern, Western, and Central.

The results imply that DTMV adoption increases productivity. The transitional heterogeneity effect is positive highlighting that the effect is bigger for the households that actually adopted DTMV compared to those that did not adopt. We also disaggregate the impact of DTMV adoption against other improved varieties and local varieties. Results show that the impact (for adopters) is higher when compared to local varieties with yields increasing by 18.9% compared to the 14% when DTMV yields are compared against other (non-DTMV) improved varieties. This compares reasonably to earlier estimates of a 25% yield advantage of improved maize varieties over local maize varieties in Africa (Smale and Jayne, 2003).

#### 4.1.2 Impact on the variance of yield

Table 4 presents the maize yield variance under actual and counterfactual conditions. The yield variance from the ESR model are used to examine the extent to which DTMV adoption stabilizes yield, one of the intrinsic characteristics of DTMVs. The treatment effects of DTMV adoption on the variance of yield are, however, only significant for non-adopters, i.e., only non-adopters would have seen their variance reduced by 2% had they adopted.

**Table 4:** Estimates of the effect of DTMV adoption on the variance of maize yield, 2015 survey, Uganda (ESR model).

Outcome variables	Household type and treatment effect	Decision stage		Effect on adoption	Chang e (%)
		To Adopt	Not to adopt		
Log of yield variance:		(1)	(2)	(3)	(4)
1. DTMV vs all non-DTMV	Adopters (ATT)	12.89	12.77	0.13	1
	Non-adopters (ATU)	12.46	12.73	−0.27^**^	−2.1
	Heterogeneity effect	0.44	0.04	0.4	3.1
2. DTMV vs improved non-DTMV	ATT	12.89	12.76	0.14	1.1
	ATU	12.46	12.72	−0.26^**^	−2.0
	Heterogeneity effect	0.44	0.04	0.4	3.1
3. DTMV vs local	ATT	12.89	12.81	0.09	0.17
	ATU	12.46	12.77	−0.31^**^	−2.4
	Heterogeneity effect	0.44	0.04	0.4	3.1

^**^p < 0.05.

#### 4.1.3 Impact on the skewness of yield

Skewness characterizes the degree of asymmetry of a distribution around its mean. It is a measure of the extent of exposure to the risk of crop failure. The skewness of maize yield and its counterfactual are presented in Table 5. The yield skewness from the ESR model are used to examine the extent to which DTMV adoption reduces exposure to downside risk, one of the intrinsic characteristics of DTMVs. The results show that DTMV adoption increases positive yield skewness, which suggests that DTMV adoption reduces the probability of crop failure. When compared against all non-DTMVs, DTMV adoption increases positive skewness by 30%, by 29% when compared with improved non-DTMVs, and by 33% when compared against local varieties.

**Table 5:** Estimates of the effect of DTMV adoption on maize yield skewness (downside risk), 2015 survey, Uganda (ESR model).

Outcome variables	Household type and treatment effect	Decision stage		Effect on adoption	Change (%)
Log of yield skewness (downside risk):		To adopt	Not to adopt		
1. DTMV vs all non-DTMV	Adopters (ATT)	14.71	11.3	3.41^***^	30.2
	Non-adopters (ATU)	13.89	14.45	−0.56	−3.9
	Heterogeneity effect	0.82	−3.15	3.97	34.1
2. DTMV vs. improved non-DTMV	ATT	14.71	11.41	3.3^***^	28.9
	ATU	13.89	14.47	−0.58	−4.0
	Heterogeneity effect	0.82	−3.06	3.88	32.9
3. DTMV vs. local	ATT	14.71	11.05	3.66^***^	33.1
	**ATU**	13.89	14.39	−0.50	−3.5
	Heterogeneity effect	0.82	−3.34	4.16	36.6

^***^p < 0.01.

#### 4.1.4 Heterogeneity effects attributed to drought incidence and severity

**Fig. 5:**
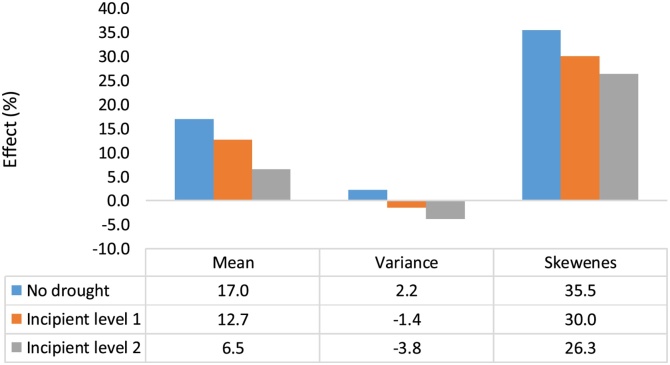
Effect of DTMV adoption with and without incipient drought, 2015 season, Uganda.

One may expect DTMV impacts to be higher when under drought. Heterogeneity effects of DTMV adoption under different drought conditions were estimated using the Drought Severity Index (DSI) as provided by Mu et al. (2013) and following Wossen et al. (2017). Table 6 depicts DSI values for all surveyed villages and results show that 72% of surveyed locations did not experience a drought in the 2015 season, while 27% experienced an incipient form of drought, and 1% experienced a mild drought – the most severe drought level observed during the survey year. Drought was thus not a major issue in the survey year for the bulk of the households. None of the estimated heterogeneity effects of adopting DTMV on the mean yield, its variance, and skewness under different drought regimes was found to be significant (Fig. 5). DTMV adoption improved the mean yield by 17% under normal rainfall conditions, and its yield benefit declined to 13% and 6.5% under incipient drought conditions (level 1 and 2, respectively). The adoption also reduced skewness by about 35.5% under normal conditions and by 26.3% under incipient drought conditions (level 2). Table 10 presents details of the switching regression results on the determinants of adoption and of yield.

**Table 6:** Drought incidence classification of survey villages for 2015 season, Uganda.

Type of drought		Drought Severity Index (DSI)	Share of villages (%)
No drought		0	72
Incipient drought	Level 1	−0.01 to −0.29	20
	Level 2	−0.3 to −0.59	7
Mild drought		−0.6 to −0.89	1
Moderate drought		−0.9 to −1.19	0
Severe drought		−1.2 to −1.49	0
Extreme drought		<−1.5	0

### 4.2 Impacts of drought-tolerant maize adoption on resource use

Table 7 presents the estimated effects of DTMV adoption on selected resource uses. DTMVs adoption increased the area planted with maize by 0.29 ha (column 1), which represents a 59% increase (column 2). Consistent with expectations, DTMV adoption reduced the recycling of maize seed. The straightforward comparison of adopters and non-adopters suggested differences in application of fertilizer, manure, and pesticides (Table 2). However, after controlling for other factors, DTMV adoption had no effect on the application of the various inputs (i.e., insignificant coefficients for DTMV adoption observed in Table 7 columns 5–8).

**Table 7:** Estimated effects of DTMV adoption on resource use, 2015 survey, Uganda (regression).

	Maize area (ha) (1)	Log (maize area) (2)	All maize seed (kg/ha) (3)	Recycled seed (kg/ha) (4)	Fertilizer- use (1=yes, 0=otherwise) (5)	Manure use (1=yes, 0=otherwise) (6)	Herbicide use (1=yes, 0=otherwise) (7)	Chemical use (1=yes, 0=otherwise) (8)
DTMV adoption	0.288^**^	0.587^**^	−4.329	−11.20^**^	0.049	0.032	−0.003	−0.021
	(0.142)	(0.204)	(3.761)	(4.79)	(0.10)	(0.038)	(0.10.05)	(0.109)
Other controls	Yes	Yes	Yes	Yes	Yes	Yes	Yes	Yes
Mean of dependent variable	0.49	−0.99	26.3	16.72	0.12	0.04	0.04	0.12
Observations	800	800	800	800	800	800	800	800

Figures in parentheses are robust standard errors; ^**^p < 0.05.

**Fig. 6:**
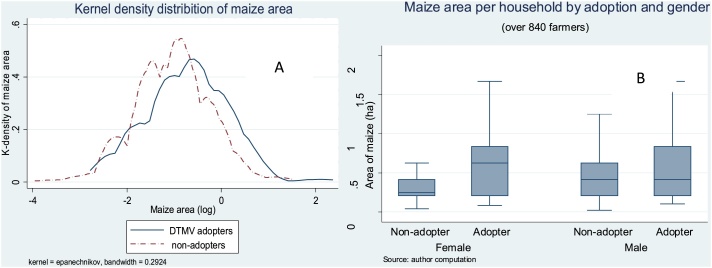
Kernel density distribution and the area of maize among DTMV adopters and non-adopters, 2015 survey, Uganda.

Fig. 6 further explores the expansion in the maize area cultivated. Panel A depicts the distribution of the maize area per household for DTMV adopters and non-adopters. The left tail of the distribution further suggests that left skewness was lower for DTMV growers than those that grew other maize varieties. The two distributions of maize areas between DTMV adopters and non-adopters were also significantly different (Kolmogorov-Smirnov test). Panel B presents the average maize area for DTMV adopters and non-adopters by gender of the household head. The average maize area is particularly low among female-headed households that did not adopt DTMV, and highest among female-headed households that adopted DTMVs.

Inadequate farm labour can be an adoption barrier to labour-intensive practices, hence potentially constraining the production system. Hired labour can complement the labour supplied by the household. The hiring of labour was quite prevalent in the study regions, with about 45% of the households reporting that they hired some labour for their maize farming activities. Table 8 presents the estimated effects of DTMV adoption on the hiring of labour in general, and for the hiring of labour at different stages of crop development (including land preparation, chemical and fertilizer application, weeding, as well as harvest and post-harvest activities). DTMVs adoption did not increase the likelihood of hiring labour, and no impacts were detected across the different activities for which labour was hired.

**Table 8:** Effects of DTMV adoption on hiring labour, 2015 survey, Uganda.

	Hired labour (1=yes, 0=Otherwise) (1)	Log of the value of all hired labour (2)	Hiring labour for land preparation (1=yes, 0=Otherwise) (3)	Hiring labour for chemical and fertilizer application and weeding (1=yes, 0=Otherwise) (4)	Hiring labour for harvest, post-harvest, transporting (1=yes, 0=Otherwise) (5)
DTMV adoption	0.266 (0.215)	7.39 (4.75)	0.203 (0.261)	0.192 (0.278)	0.088 (0.196)
Other controls	Yes	Yes	Yes	Yes	Yes
Mean of dependent variable	0.45	1.24	0.38	0.38	0.30
Observations	840	840	840	840	840

DTMV adoption appeared to encourage mechanization through the hiring of ox-ploughs and maize threshers (Table 9). These findings suggest that DTMV adoption has a crowding-in effect on the utilization of mechanized tools for maize production and processing, with the thresher likely associated with the yield increase. Nonetheless DTMV adoption had an insignificant effect on land hiring and the overall total value of assets hired by the household. The earlier reported expansion in the maize area cultivated associated with DTMV adoption is thus primarily on existing own farm land.

**Table 9:** Effects of DTMV adoption on the hiring of other assets, 2015 survey, Uganda.

	Log of value of all assets (1)	Log of value of land rented (2)	Log of value of hired oxen (3)	Log of value of hired ox plough (4)	Log of value of thresher (5)
DTMV adoption	2.73 (3.0)	0.608 (3.54)	4.87 (2.65)	4.71^*^ (2.53)	5.83^**^ (2.34)
Other controls	Yes	Yes	Yes	Yes	Yes
Mean of dependent variable	−0.28	−3.12	−3.07	−3.97	−5.16
Observations	840	840	840	840	840

^*^p < 0.1, ^**^p < 0.05.

**Table 10:** Full information maximum likelihood estimates of the switching regression model.

			FIML endogenous switching regression of determinants of yield			
Variable	Selection equation of the determinants of adoption		Adoption = 1 (adopters)		Adoption = 0 (non-adopters)	
	Coeff	SE	Coeff.	Se	Coeff.	Se
Use of hired labor (dummy, 1=yes, 0=otherwise)	0.31^**^	0.11	0.29^*^	0.17	0.15^**^	0.06
Use of fertilizer (dummy)	0.21	0.18	0.73^***^	0.20	0.12	0.10
Use of chemicals (dummy)	0.01	0.18	0.09	0.18	0.18^**^	0.09
Use of herbicides (dummy)	0.48	0.30	0.27	0.34	0.09	0.15
Use of manure (dummy)	0.57^**^	0.25	0.52	0.31	−0.05	0.17
Fertile soil (dummy)	0.19	0.12	0.08	0.15	0.13^**^	0.05
No erosion (dummy)	−0.21	0.16	−0.04	0.21	0.00	0.08
Irrigated crop (dummy)	−0.65	0.41	0.34	0.56	0.12	0.18
Years of education for head of household	0.00	0.02	−0.04^**^	0.02	0.01	0.01
Household size	0.06^***^	0.02	0.03	0.03	0.00	0.01
Group membership (dummy)	0.06	0.14	0.28	0.16	−0.01	0.05
Income allows buildup of savings (dummy)	0.96^***^	0.28	0.77	0.52	0.27^**^	0.13
Income allows to save a little (dummy)	0.87^***^	0.27	0.45	0.47	0.04	0.10
Income equal expenses (dummy)	0.39	0.28	0.02	0.40	−0.01	0.09
Income insufficient to make savings (dummy)	0.80^***^	0.28	0.05	0.48	0.08	0.11
Plot on steep slope (dummy)	−0.28	0.26	0.12	0.31	−0.03	0.11
Plot on moderate slope (dummy)	−0.09	0.16	−0.04	0.19	0.06	0.07
Age of the head of household (yrs)	−0.14	0.19	0.28	0.23	−0.12	0.08
Eastern region	0.95^***^	0.33	−1.34^**^	0.54	−0.53^***^	0.12
Western Region	1.30^***^	0.37	−0.88	0.65	−0.18	0.19
Northern region	0.23	0.34	−1.37^***^	0.47	−0.49^***^	0.10
Received information on varieties (dummy)	0.22^**^	0.11				
_cons	−2.37^***^	0.80	6.31^***^	1.33	7.53^***^	0.30
/lns1	−0.29	0.28				
/lns2	−0.37^***^	0.03				
/r1	0.64	0.70				
/r2	−0.20	0.47				

Figures in parenthesis indicate the standard errors; ^***^p < 0.01, ^**^p < 0.05, ^*^p < 0.1.

## 5 Conclusions and implications

This paper evaluates the impacts of the adoption of Drought Tolerant Maize Varieties (DTMVs) on productivity, yield variance and exposure to down side risk using cross-sectional farm household level data from Uganda. The study further evaluates the extent to which DTMV adoption crowds-in additional investments to maize farming. The causal impact of adopting DTMV is estimated by utilizing an endogenous switching regression, while the impact on crowding-in additional investments is estimated using a special regressor approach.

DTMV adopters and non-adopters differ in a number of socioeconomic characteristics. Overall, DTMV adopters appear to be better endowed, cultivate more maize and have higher input use and yields. However, after controlling for adopter characteristics, DTMV adoption increased yield by 15% and reduced exposure to crop failure by 30%. The results confirm the potential role of DTMVs as a technology that can potentially mitigate against the negative impact of adverse rainfall conditions, set to be increasingly relevant with climate change. Moreover, we found DTMV adoption to crowd-in investments in maize production at the extensive margin through the expansion of the maize area, and to a limited extent on the intensive margin through increased mechanization of the land preparation process and threshing.

In our sample, 14% of the households reported having planted at least one DTMV in one of their maize plots. Given the generally positive impacts of DTMV, the question arises why DTMVs are not adopted more extensively – a question shared by Asfaw et al. (2012). Earlier work by Simtowe et al. (in press) using the same survey data, identified information and seed constraints as the key hurdles to the further diffusion of DTMVs in Uganda. The results from our study confirm the robust benefits from DTMV adoption in Uganda. This calls for a concerted effort to alleviate the key constraints and to enable the further uptake and scaling of these promising DTMVs among farming communities as a productivity enhancement, as well as drought risk reduction technology. Moreover, the fact that DTMV adoption crowds in investments into maize farming at the extensive margin, provides an opportunity for scaling maize production in Uganda and transforming its maize sector.

## Footnotes

1 Feed the Future is the US government’s global hunger and food security initiative that addresses the root causes of poverty, hunger and undernutrition. It transforms the agricultural sector – from farms to markets to tables – through country-led priorities and partnerships with governments, donor organisations, the private sector and civil society.

2 We use the concept “diffusion” to imply awareness or knowledge of the DTMVs by the farmers. In the adoption literature, however, the terms “diffusion” and “adoption” are mostly used interchangeably (Rogers, 1976; Sunding and Zilberman, 2001). Feder et al., (1985) describes technology adoption as a multistage process the decision-maker undergoes from the time they get exposed to the technology through to the time they decide to start using the DTMVs.
