# Supplementary material for: Impacts of drought-tolerant maize varieties on productivity, risk, and resource use: Evidence from Uganda
Source: Land use policy. 2019 Nov;88:104091. doi: 10.1016/j.landusepol.2019.104091 (PMC6894317; doi:10.1016/j.landusepol.2019.104091)
Supplement: Supplementary file 1 [file mmc1.docx]

# Appendix

## The effects on productivity, variance, and skeweness

A farmer chooses to adopt ($A_{i}$ = 1) if $A^{*}>0,$0 otherwise, where $A^{*}$ represents the expected benefits of adoption compared to non-adoption. The selection equation for the latent variable ($A_{i}^{*}$) is specified as follows;

|  | $A_{i}^{*}=f\left( m,x,v,w,z,\gamma\right)+\mu_{1}$ with $A=1\{A_{i}^{*}>0$ | (1) |
| --- | --- | --- |

where $m$ refers to adoption of DTMVs, $x$ represents inputs other than DTMV seed, $v$ refers to a vector of variables of socio-economic, farm and social capital, $w$ is a vector of random variables representing uncontrollable factors affecting maize output such as rainfall, $z$ is instrument variables and $\gamma$ represents a vector of parameters for estimation.

In order to account for selection bias, the ESR outcome equation conditional to DTMVs adoption - two regimes faced by farmers is specified as follows:

|  | $Regime 1: Y_{1i}= f\left( m,x,v,w,\beta_{1} \right)+\varepsilon_{1i}$ if $A_{i}=1$ | (2) |
| --- | --- | --- |
|  | $Regime 2: Y_{2i}= f\left( m,x,v,w,\beta_{2} \right)+\varepsilon_{2i}$if $A_{i}=0$ | (3) |

where $Y_{1i}$and $Y_{2i}$ are the dependent variables in the two continuous equations representing the yield of DTMVs adopters and non-adopters respectively. $\varepsilon_{1i}$ and $\varepsilon_{2i}$ are error terms of the outcome variables and $\beta_{1}$ , $\beta_{2}$ are vectors of parameters to be estimated.

The error terms ($\mu_{1}$, $\varepsilon_{1i} and \varepsilon_{2i} )$ in both the selection equation and outcome equations (1, 2 and 3) are assumed to have a trivariate normal distribution, with zero mean and covariance matrix (Ω) as specified in the below equation:

| $\sigma_{e1}^{2}$   \| $.$ \| \| --- \| | $.$   \| $\sigma_{e2}^{2}$ \|  \| \| --- \| --- \| | $\sigma_{e2u}$ |
| --- | --- | --- | --- | --- | --- |
| . | .   \|  \|  \| \| --- \| --- \| | $\sigma_{u}^{2}$ |

$$\sigma_{e1u}$$

$cov(e_{1i},e_{2i}, u_{i})=$

where $\sigma_{u}^{2}$ is a variance of the error term in the selection equation (1 ), $\sigma_{e1}^{2}$ and $\sigma_{e2}^{2}$ are variances of the error terms ($\varepsilon_{1i} and \varepsilon_{2i})$in the continuous outcome equations (2) and (3), $\sigma_{e1u}$ is the covariance of ${(\mu}_{1}$, ${e1}_{i})$ while $\sigma_{e2u}$ is the covariance of ${(\mu}_{1}$, $\varepsilon_{e2i})$. The covariance between $\varepsilon_{1i}$and $\varepsilon_{2i}$ are not defined since $Y_{1i}$ and $Y_{2i}$are not observed simultaneously.

## Conditional expectations, treatment and heterogeneity effects

The Endogenous Switching Regression model (ESR) estimates allow comparison of the expected productivity gain and risk exposure reduction of adopters of DTMVs with respect to non- adopters; and allow further examination into the expected productivity gain and risk exposure reduction in the counterfactual hypothetical cases that the adopters did not adopt or that the non-adopters adopted DTMVs (Asfaw et al., 2012). Table 1 presents conditional expectations for the outcome variables in the four cases as expressed above, and whose mathematical expressions are presented in Asfaw et al., (2012) and Wossen *et al.,* (2017):

|  | $E(Y_{1i}\vert A_{i}=1)= f\left( m,x,v,e,w,\beta_{1} \right)+\lambda_{1i}\sigma_{1\mu}$ | (1) |
| --- | --- | --- |
|  | $E(Y_{2i}\vert A_{i}=0)= f\left( m,x,v,e,w,\beta_{2} \right)+\lambda_{2i}\sigma_{2\mu}$ | (2) |
|  | $E(Y_{2i}\vert A_{i}=1)= f\left( m,x,v,e,w,\beta_{2} \right)+\lambda_{1i}\sigma_{2\mu}$ | (3) |

|  | $E(Y_{1i}\vert A_{i}=0)= f\left( m,x,v,e,w,\beta_{1} \right)+\lambda_{2i}\sigma_{1\mu}$ | (4) |
| --- | --- | --- |

Cases (a) and (b) in Table 1 are the actual expectation observed in the sample. Cases (c) and (d) are counterfactual outcomes. The effect of the treatment “To adopt” on the treated (ATT) is estimated as the difference between (a) and (c).

|  | $ATT=E(Y_{1i}\vert A_{i}=1)- E(Y_{2i}\vert A_{i}=1)$ | (5) |
| --- | --- | --- |

Which represents the effect of adopting DTMVs on the yield of adopters. Similarly, the effect of the treatment on the untreated (ATU) for the non-adopter households is calculated as the difference between (d) and (b):

|  | $ATU=E(Y_{1i}\vert A_{i}=0)- E(Y_{2i}\vert A_{i}=0)$ | (6) |
| --- | --- | --- |

Heterogeneity effects were further estimated from the expected outcome equations described in 13-16. Following Di Falco et al., (2011) the base heterogeneity effect for adopters of DTMVs (${BH}_{1}$) is the difference between (a) and (d) and expressed as follows:

| $BH_{1}=E(Y_{1i}\vert A_{i}=1)- E(Y_{1i}\vert A_{i}=0)$ | (7) |
| --- | --- |

The difference between (c) and (b) measure the effect of base heterogeneity (${BH}_{2}$) for non-adopters and it is expressed as follows:

|  | $BH_{2}=E(Y_{2i}\vert A_{i}=1)- E(Y_{2i}\left\vert A_{i}=0 \right)$ | (8) |
| --- | --- | --- |

The “transitional heterogeneity” (TH) which measures whether the effect of adopting DTMVs is larger or not for the actual DTMVs adopters or for non-adopters in they adopted; (ie the difference between equations 5 and 6) is expressed as:

|  | $TH=ATT-ATU$ | (9) |
| --- | --- | --- |

Table 1: Conditional expectations, treatment and heterogeneity effects

| **Sub-groups** | Decision stage | | |
| --- | --- | --- | --- |
|  | To adopt | Not to adopt | Treatment effects |
| DTMV adopters | $\left( a \right)E(Y_{1i}\vert A_{i}=1)$ | $\left( c \right)E(Y_{2i}\vert A_{i}=1)$ | $ATT$ |
| DTMV non adopters | $\left( d \right)E(Y_{1i}\vert A_{i}=0)$ | $\left( b \right)E(Y_{2i}\vert A_{i}=0)$ | $ATU$ |
| Heterogeneity effects | ${BH}_{1}$ | ${BH}_{2}$ | $TH$ |

As the objectives of the study includes understanding the effects of |DTMV adoption on variance (second moment) and skewness (third moment), the ATT and ATU for second moment of maize yield (variance) was defined as follows:

|  | $ATT=E(\mu_{1i}^{2}\vert A_{i}=1)- E(\mu_{2i}^{2}\vert A_{i}=1)$ | (10) |
| --- | --- | --- |
|  | $ATU=E(\mu_{1i}^{2}\vert A_{i}=0)- E(\mu_{2i}^{2}\vert A_{i}=0)$ | (11) |

We further estimate ATU and ATT estimates for skeweness as:

|  | $ATT=E(\mu_{1i}^{3}\vert A_{i}=1)- E(\mu_{2i}^{3}\vert A_{i}=1)$ | (12) |
| --- | --- | --- |
|  | $ATU=E(\mu_{1i}^{3}\vert A_{i}=0)- E(\mu_{2i}^{3}\vert A_{i}=0)$ | (13) |

## Effects on resource use

In order to assess crowding-in effects of DTMV adoption on resource use in maize farming, we employ the approach proposed by Emerick *et al*., (2016) with the following specification:

$y_{i}=\beta_{0}+\beta_{1}{treatment}_{i}{+\beta_{2}x_{e}+\varepsilon}_{i}$ (14)

where $y_{i}$is an outcome observed for farmer $i$ and $\varepsilon_{i}$ is the error term. The analysis is at plot level; hence we use a plot level treatment indicator for outcomes at plot level. The estimate of $\beta_{i}$represents an average effect of the treatment while $x_{e}$ is an endogenous regressor, and $\beta_{2}$ the correlation coefficient of endogenous regressor and the dependent outcome variable. The approach has been used by Maccini and Yang (2009) to investigate how early-life shocks affect outcomes later in life. However, as the selection into treatment is non-random, we apply a special regressor method proposed by Lewbel et al., (2012).
